# Image dataset for benchmarking automated fish detection and classification algorithms

**DOI:** 10.1038/s41597-022-01906-1

**Published:** 2023-01-03

**Authors:** Marco Francescangeli, Simone Marini, Enoc Martínez, Joaquín Del Río, Daniel M. Toma, Marc Nogueras, Jacopo Aguzzi

**Affiliations:** 1 Electronics Department, Polytechnic University of Catalonia (UPC), Vilanova i la Geltrú, Barcelona, 08800 Spain; 2 Institute of Marine Sciences, National Research Council of Italy, La Spezia, Italy; 3 Stazione Zoologica Anton Dohrn (SZN), Naples, 80127 Italy; 4 European Multidisciplinary Seafloor and Water Column Observatory, Rome, Italy; 5 Department of Marine Renewable Resources, Institute of Marine Science (ICM-CSIC), Barcelona, 08016 Spain

**Keywords:** Time-lapse imaging, Marine biology, Ecosystem ecology, Population dynamics

## Abstract

Multiparametric video-cabled marine observatories are becoming strategic to monitor remotely and in real-time the marine ecosystem. Those platforms can achieve continuous, high-frequency and long-lasting image data sets that require automation in order to extract biological time series. The OBSEA, located at 4 km from Vilanova i la Geltrú at 20 m depth, was used to produce coastal fish time series continuously over the 24-h during 2013–2014. The image content of the photos was extracted *via* tagging, resulting in 69917 fish tags of 30 taxa identified. We also provided a meteorological and oceanographic dataset filtered by a quality control procedure to define real-world conditions affecting image quality. The tagged fish dataset can be of great importance to develop Artificial Intelligence routines for the automated identification and classification of fishes in extensive time-lapse image sets.

## Background & Summary

In a context of global climate change and increasing human impact in coastal marine areas, the monitoring of changes in fish behaviour and population abundances is becoming strategic to provide data on ecosystem productivity, functioning and derived services (e.g., the status of already overexploited stocks)^1–3^. For this reason, monitoring the temporal dynamics of fish communities is of pivotal importance to distinguish the variability in species composition, due to diel and seasonal activity rhythms, from more long-lasting trends of change^4,5^. The temporal trend of fish presence and abundance, obtained from the analysis of imagery data, is produced by the rhythmic migration of populations into the marine 3D space seabed and water column scenario^6–8^. The information derived from such dynamics coupled with environmental (oceanographic and meteorological) data provide useful information regarding species ecological niche^9–11^, and allow understanding and forecasting the impact of anthropic activities (e.g., commercial fishing, urban and port expansion) and the consequent mitigation actions (e.g., establishment of marine protected areas)^7,12,13^.

Cabled video-observatory monitoring technology is considered as the core of growing *in situ* and robotized marine ecological laboratories in coastal and deep-sea areas^14,15^. International initiatives about marine observatories infrastructures, like for example the European Multidisciplinary Seafloor and water column Observatory (EMSO-ERIC), the Joint European Research Infrastructure of Coastal Observatories (JERICO-RI), or the Ocean Network Canada (ONC) are becoming widespread all over the world^16^, and increasingly install multiparametric sensors that, beside the imaging depicting biological information, also acquire oceanographic and geo-chemical data^13,17^.

Unlike other types of data, the scientific content of videos and images is not immediately usable. To overcome this problem, the image content is often inspected by trained operators in order to manually extract relevant biological information, such as the number of individuals and the corresponding classification into species^18–20^. This manual process requires a considerable human effort, and it is really time demanding. For this reason, automated image analysis methodologies for the extraction and coding of the image content need to be urgently defined and developed in order to transform imaging devices into actual biological tools for the underwater observing systems^21,22^.

This article describes a dataset of underwater images suitable for studying, developing and testing methodologies for automated image analysis. The images were acquired at the seafloor cabled multiparametric video-platform “Observatory of the Sea” (OBSEA; www.obsea.es), located in a fishing protected area, 20 m depth, 4 km off the Vilanova i la Geltrú coast, near Barcelona (Spain)^23,24^. The image dataset consists of 33805 images containing 69917 manually tagged fish specimens, acquired every 30 minutes over day and night, during two consecutive years (i.e., from 1^st^ January 2013 to 31^st^ December 2014). The dataset encompasses and replicates the most relevant seasonal dynamics of environmental change affecting fish species abundance and assemblage at the study site^25^. In fact, coastal fish physiology and behaviour are highly responsive to changes in photo-period (i.e., light intensity and photophase duration)^26^, nutrients and pollutants^27,28^ and oceanographic regimes (i.e., currents, temperature, and salinity)^29–31^. Thus, OBSEA monitoring area represents a real-world operational context common to many other temperate coastal underwater observing systems.

Together with the image dataset, we also provided oceanographic and meteorological time series, whose readings have been averaged and recorded synchronously with time-lapse images. Those data are for water temperature, change in depth, salinity, air temperature, wind speed and direction, solar irradiance and water precipitation. We added those environmental time series as contemporarily acquired, in order to provide a quality aspect to the real-time world context of image acquisition, to be used as metrics for image processing efficiency^32^. Moreover, the use of those data has been of relevance to provide hints in cause-effect studies linking fish presence and behaviour upon changing environmental conditions, being already successfully exploited for automated fish recognition^32^, and for studying the temporal modulation of the species niches^33,34^.

The manually tagged fish individuals for each image make the dataset a valuable benchmark for the multidisciplinary marine science community consisting of biologists, oceanographers, and a growing community of computer scientists and mathematicians skilled in Artificial Intelligence and data science. Methodological comparison could be not only specifically conceived for fish detection and classification, such as Fish4Knowledge^35^, but also for the emerging approaches for active and incremental learning^36–38^, or for techniques aimed at mitigating the “Concept Drift” phenomenon, when the classification performance drop for varying species assemblages at changing environmental conditions and training need to be updated^39–42^.

Finally, the reported dataset of labelled images is worthwhile for global image repositories that aim to reduce annotation effort, such as Fathomnet^43^, and, thanks to the tags and the bounding boxes associated to each individual, it can be easily split into training, validation, and test subsets (e.g., K-fold Cross-validation) in order to fit the needs of the specific image analysis algorithm used on the image dataset^32,42,44–47^.

## Methods

### OBSEA video-image underwater platform and routine

**Fig. 1:**
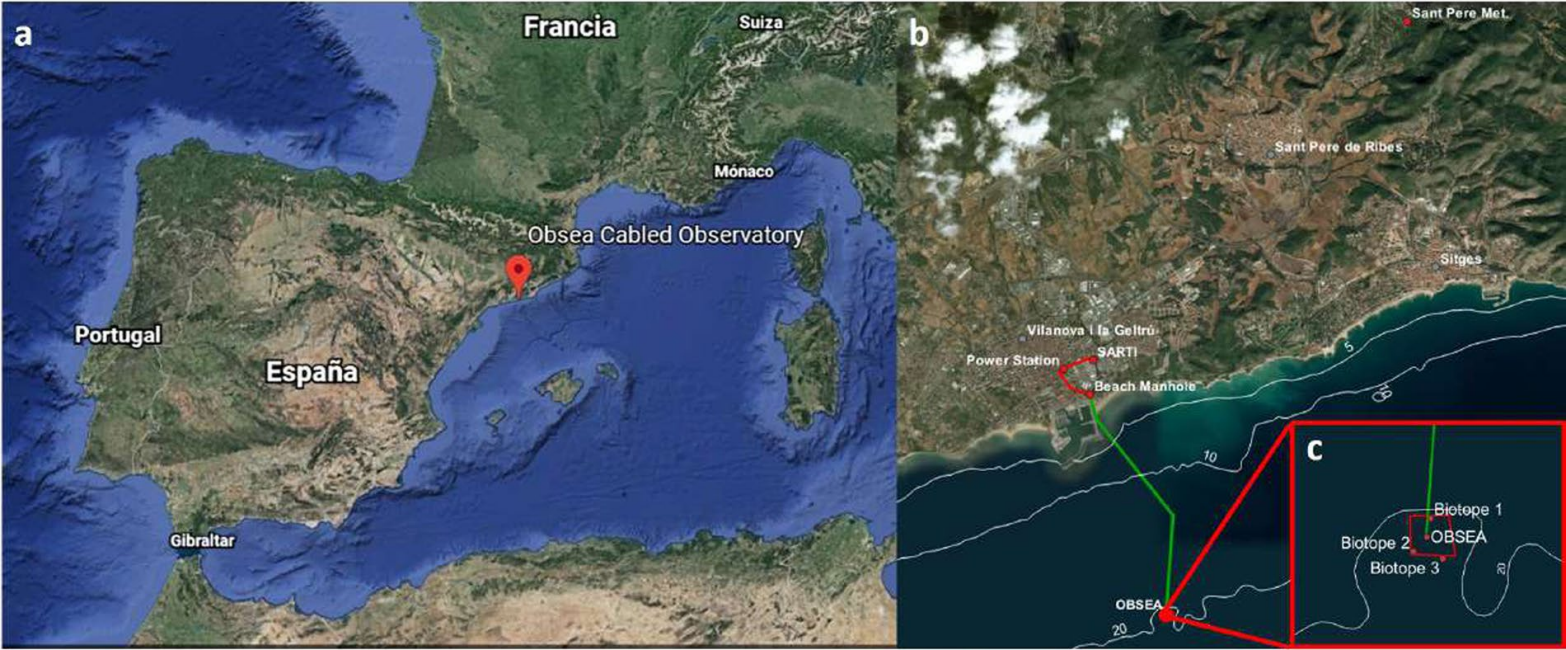
Location of the OBSEA video platform in the North-Western (NW) Mediterranean. The figure indicates the “Development Centre of Remote Acquisition and Information Processing” (SARTI) and the Sant Pere de Ribes Meteorological Station (Sant Pere Met.) positions relative to the Catalan coasts (**a**), indicating also the OBSEA position off the harbour of Vilanova i la Geltrú (**b**). Power and broadband Ethernet communications are provided to OBSEA through an underwater cable from the SARTI building (green and red tracks). The OBSEA platform is surrounded by three biotopes (**c**) and focusing on one of them (Biotope 1, c).

The OBSEA seafloor cabled observatory was deployed in 2009 within a Natura 2000 marine reserve, named “*Colls i Miralpeix*”, at 20 m depth and at 4 km off Vilanova i la Gertrú harbour (i.e., the Catalan coast of the NW Mediterranean, Spain: 41°10′54.87″N and 1°45′8.43″E) (Fig. 1). The cable observatory is located on a mixed sand and seagrass meadows (*Posidonia oceanica*) bed, being surrounded by artificial concrete reefs, deployed to protect the area from illegal trawling^23,24^.

**Fig. 2:**
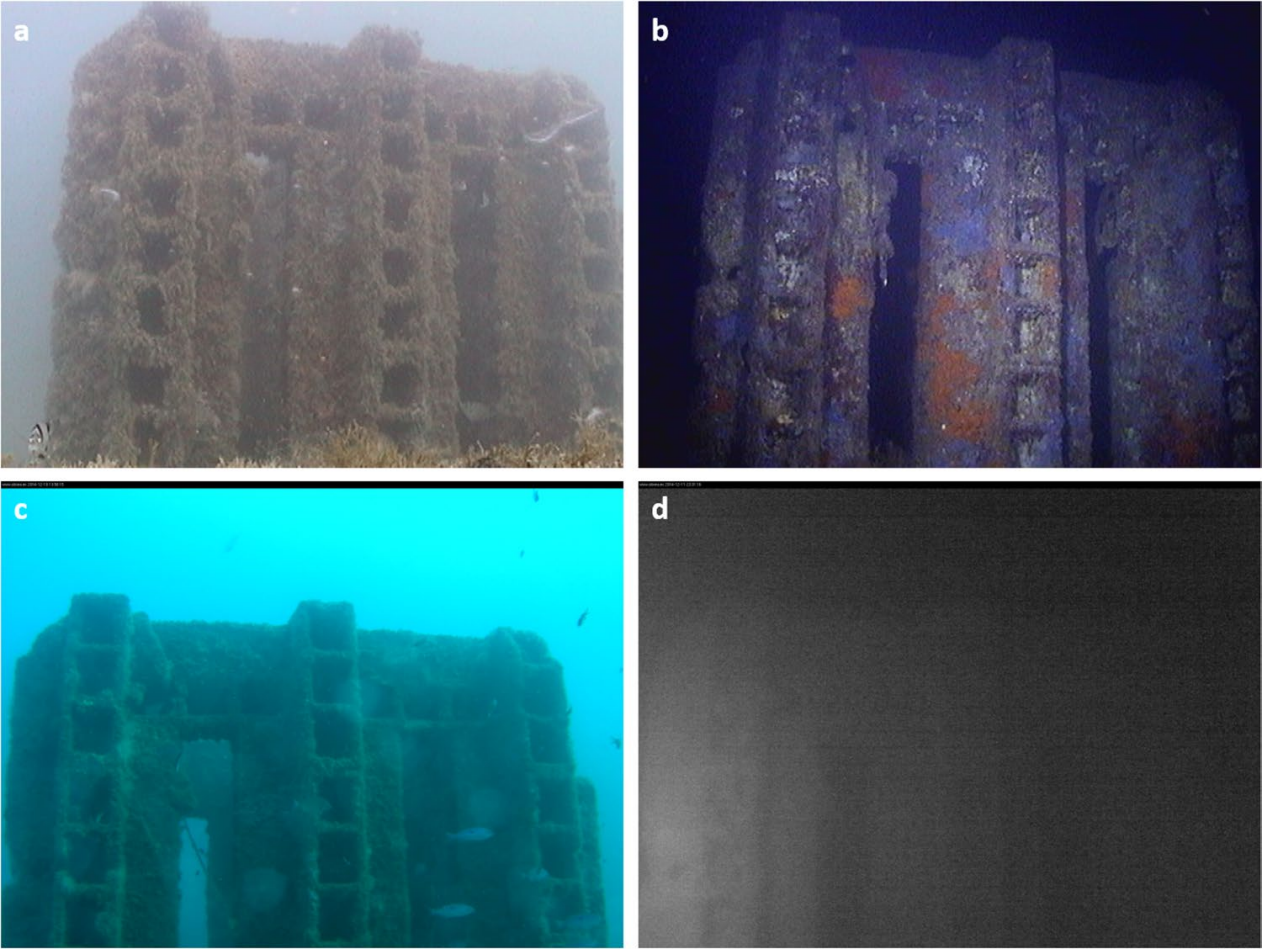
**Examples of photos acquired by the different cameras used at the OBSEA.** The Sony SNC-RZ25N (CAM1) (**a**,**b**) and the Axis P1346-E (CAM2) (**c**,**d**) cameras’ acquired photos during day and night.

The OBSEA node structure has a size in terms of width, height, and length of 1x2x1 m, respectively, with an overall weight of 5 tons. The observatory is equipped with a camera approximately at 3.5 m distance from one of these artificial reefs, with a Field of View (FOV) area of about 3 × 3 m, resulting in a 10.5 m^3^ of imaged volume (Fig. 2).

The image monitoring was performed in a 30 min time-lapse mode, by synchronising illumination at nighttime at the moment of shooting. To shoot photos at night, the camera was associated with two illuminators located beside the camera at 1 m distance from each other, each one consisting of 13 high-luminosity white LEDs. The lights were emitting 2900 lumens, with a colour temperature of 2700 kelvin and an illumination angle of 120°. An automated protocol, controlled by a LabView application, switched on-and-off the lights before and after the camera shooting, resulting in a 30 s light-on period, to allow the lights to warm up and attain the maximum amount of homogeneous illumination.

Two different cameras were used during the monitoring period: an OPT-06 Underwater IP Camera (Sony SNC-RZ25N) from 1^st^ January 2013 to 11^th^ December 2014, and an Axis P1346-E Camera thereafter until 31^st^ December 2014 (Table 1). The selected resolution of images for the first cameras was 640 × 480 pixels, whereas the second camera image resolution was 2048 × 1536 pixels (Fig. 2). The acquired images have a JPEG format for both cameras.

**Table 1:** Technical characteristic of the two cameras used for the monitoring at the OBSEA.

	Sony SNC-RZ25N (CAM1)	Axis P1346-E (CAM2)
**N. of Pixels**	3.8 MP	3 MP
**Varifocal**	4.1–73.8 mm	3.5–10 mm
**Pan Angle**	−170°–170°	72°-27°
**Tilt Angle**	−90° - 30°	/
**Focal Length-Aperture ratio**	F1.4	F1.6
**Light Sensitivity**	0.7 lux	0.5 lux
**Day-Night Function**	Yes	Yes
**Infrared Filter**	Yes	Yes
**Zoom**	18x	Digital Zoom
**Image Sensor**	1/4 type CCD Imager	CMOS RGB of progressive scan 1/3”
**Obturation Speed**	/	1/35500 - 1/6 sec
**Image Size**	640 × 480, 480 × 360, 384 × 288, 320 × 240, 256 × 192, 160 × 120	from 2048 × 1536 to 160 × 90

Technical characteristics of the two cameras (i.e., Sony SNC-RZ25N and Axis P1346-E) used between 2013–2014 at the OBSEA platform: number of pixels (N. of Pixels), varifocal, pan and tilt angle, focal length-aperture ratio, light sensitivity, presence/absence of the day-night filter, zoom, image sensor, obturation speed, and size of the saved images.

### Fish tags and annotation procedure

**Fig. 3:**
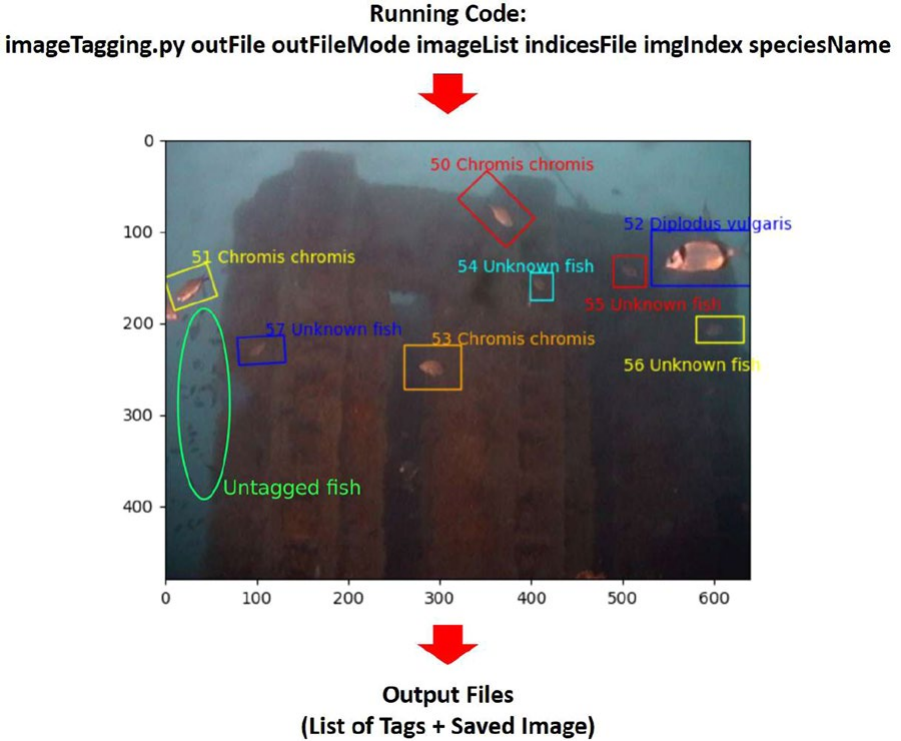
Flowchart for the tagging procedure. The tagging procedure of the photos were carried out with a Python code, at the end of which it releases as output a list of tags in text format and save the images with their bounding boxes (rectangles of different colours). Here, we report an example of a processed photo with tagged specimens and untagged fishes (green circle).

In order to tag the relevant biological content of the images (i.e., fish individuals), a Python code was developed based on the OpenCV framework for Python (https://opencv.org/)^48^ (Fig. 3).

The script allowed tracing a line around the biological subjects, calculating afterwards a bounding box (bbox). The script and all the instructions of the tagging procedure are available through the Zenodo repository^49^.

The species classification was performed according to FISHBase^50^. In those cases where the fish was not fully classifiable because too distant or badly positioned within the FOV we classified them as “Unknown fish”. This is because these unclassified fishes are important for the estimate of fish biomass (Fig. 3). Some examples deal with individuals appearing in the photo like dots. Other examples deal with overlapping fishes, such as when they form schools.

### Oceanographic and meteorological data acquisition and processing

The OBSEA was equipped with a CTD probe to measure the water temperature, salinity, and the changes of depth, calculated from shifts in water pressure (as proxy for tides). During the period between 2013–2014, two CTD probes were sequentially deployed to avoid data gaps during sensor maintenance operations (Table 2). In Table 3 the deployment periods of both CTD probes are depicted.

**Table 2:** Technical characteristics of the two CTD probes, and of the two meteorological stations.

		Range	Accuracy	Stability	Resolution	Time of Acquisition
**SBE 37-SMP**	**Conductivity**	0–7 S/m	0.0003 S/m	0.0003 S/m per month	0.00001 S/m	10 sec
	**Temperature**	−5 °C–35 °C	0.002 °C	0.0002 °C per month	0.0001 °C	10 sec
	**Pressure**	20–7000 m	0.1% of full-scale range	0.05% of full-scale range per year	0.002% of full-scale range	10 sec
**SBE 16plus V2**	**Conductivity**	0–9 S/m	0.0005 S/m	0.0003 S/m per month	0.00005 S/m	10 sec
	**Temperature**	−5 °C–35 °C	0.005 °C	0.0002 °C per month	0.0001 °C	10 sec
	**Pressure**	20–7000 m	0.1% of full-scale range	0.1% of full-scale range per year	0.002% of full-scale range	10 sec
**UPC Weather Station (Station 1)**	**Air Temperature**	−40 °C–65 °C	0.3 °C	/	0.1 °C	10 sec
	**Wind Speed**	0–322 km/h	3 km/h	/	1 km/h	10 min
	**Wind Direction**	0–360°	3°	/	1°	10 min
**Sant Pere de Ribes Weather Station(Station 2)**	**Solar Irradiance**	0–5000 W/m	typ. <3%, 5% maximum	/	1 W/m2	10 min
	**Rain**	0–20 mm/min	0.1 mm	/	0.001 mm	10 min

Technical characteristic of the two CTD sensors (i.e., SBE16 and SBE37) installed at the OBSEA, the meteorological station of the Polytechnic University of Catalonia (UPC) in Vilanova i la Geltrú (i.e., Station 1), and the meteorological station of Sant Pere de Ribes (i.e., Station 2) present during the period between 2013–2014.

**Table 3:** Deployment periods of the CTD sensors of the OBSEA.

Sensor	Deployment	Recovery
SBE 16plus V2	*2013-01-09*	*2013-04-10*
SBE 37-SMP	*2013-04-10*	*2013-04-19*
SBE 16plus V2	*2013-04-19*	*2013-12-05*
SBE 37-SMP	*2013-12-05*	*2014-03-20*
SBE 16plus V2	*2014-03-20*	*2014-09-12*
SBE 37-SMP	*2014-09-12*	*2014-12-31*

Details of the deployment and recovery of the CTD probes during the period between 2013–2014.

Moreover, meteorological variables were measured from the meteorological station on the roof of the Polytechnic University of Catalonia (UPC) building in Vilanova i la Geltrú, and from the meteorological station of Sant Pere de Ribes, Spain (www.meteo.cat) (Table 2). The first one was a Vantage Pro2 meteorological station. This station was installed to collect data on the air temperature, wind speed and direction. Furthermore, we compiled data for solar irradiance and rain from the meteorological station in Sant Pere de Ribes. This station was equipped with a Pyranometer SKS 1110 to measure solar irradiance, and a Rain[e] sensor for the rain.

All the oceanographic and meteorological data were averaged every 30 min, in order to have mean and standard deviation measurements contemporary to the timing of all acquired images (see above), except for the irradiance and rain, that were compiled selecting and extracting only readings correspondent to the acquired image timings (see above).

In order to filter these data, we applied a Quality Control (QC) procedure for all the environmental variables except for the solar irradiance and rain, considered prefiltered and institutional data. This procedure is based on the guidelines from the Quality Assurance of Real-Time Oceanographic Data (QARTOD), issued by the United States Integrated Ocean Observing System (US-IOOS) Program Office, as part of its Data MAnagement and Cyberinfrastructure (DMAC) (https://ioos.noaa.gov/project/qartod/). This QC procedure was based on the IOOS QC python tools (https://github.com/ioos/ioos_qc). Following the QARTOD guidelines, the following tests were applied:

- Gross Range test. Highlight data points that exceeded sensors or operator selected minimum and maximum levels.
- Climatology test. Data points that fall outside the seasonal ranges introduced by the operator.
- Spike test. Data points n-1 that exceeded a selected threshold relative to adjacent points.
- Rate of change test. Examination of excessive rises or falls in the data.
- Flat line test. Examination of invariant values in the data.

Each time that the quality test was run, each value of the dataset was flagged with a quality control code. The QC flags and meanings are shown in Table 4.

**Table 4:** Quality control flags’ codes and meanings.

Flag Value	Flag Meaning
1	*Good Data*
2	*QC Not Applied*
3	*Suspicious Data*
4	*Bad Data*
9	*Missing Data*

Quality control flags values and respective meanings applied to the environmental data.

The oceanographic and meteorological data were annotated into comma delimited files (CSV) with additional information on QC flags, time stamps, and measurement devices used for their acquisition^51–53^.

## Data Records

### Tagging outputs

**Fig. 4:**
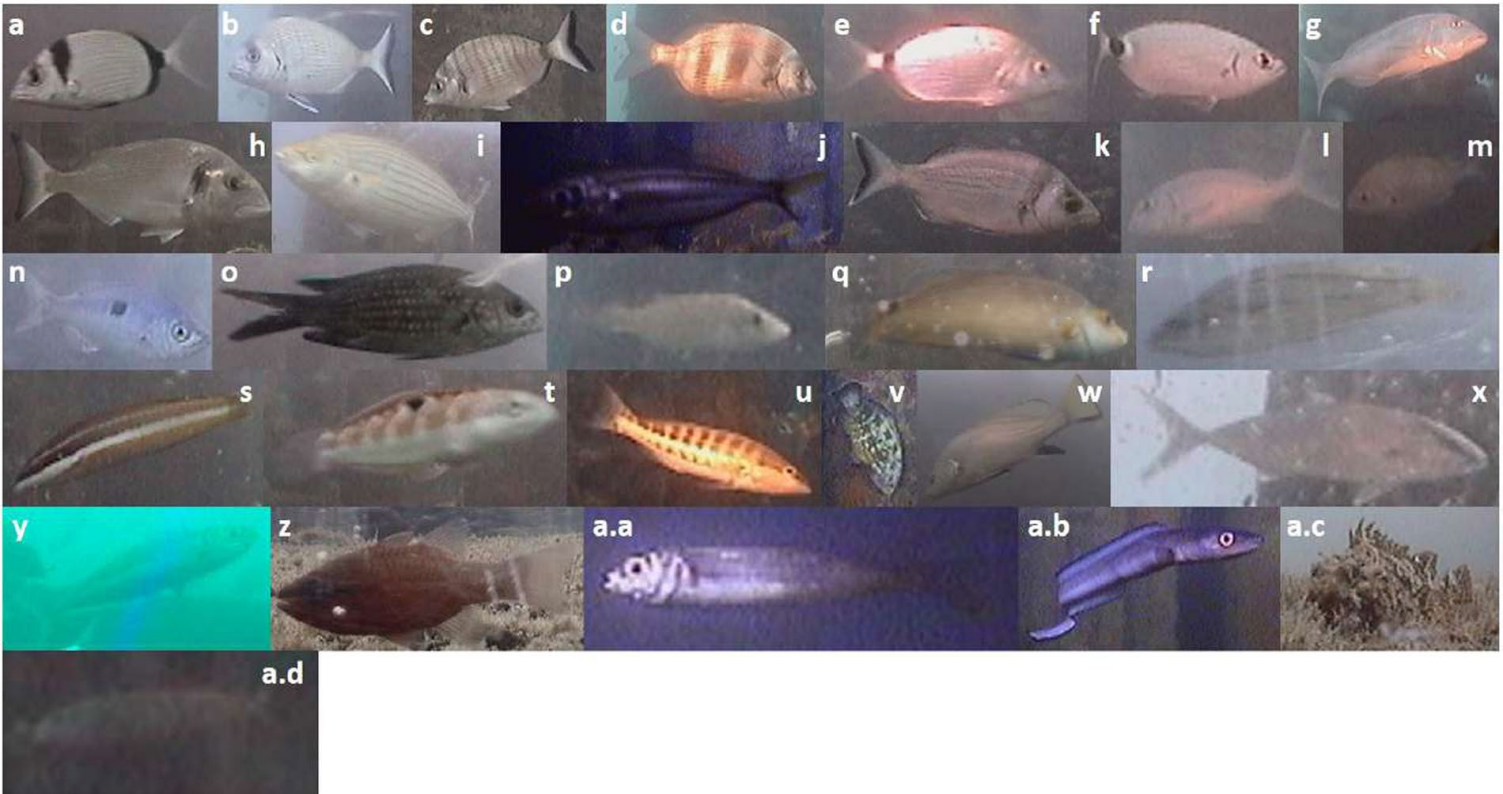
Photomosaic of the fish taxa encountered during the tagging procedure. Examples of photos of the 29 fish taxa recognized during the tagging, plus an example of an unclassified fish: (**a**) *Diplodus vulgaris*, (**b**) *Diplodus sargus*, (**c**) *Diplodus puntazzo*, (**d**) *Diplodus cervinus*, (**e**) *Diplodus annularis*, (**f**) *Oblada melanura*, (**g**) *Dentex dentex*, (**h**) *Sparus aurata*, (**i**) *Sarpa salpa*, (**j**) *Boops boops*, (**k**) *Spondyliosoma cantharus*, (**l**) *Pagrus pagrus*, (**m**) *Pagellus* sp., (**n**) *Spicara maena*, (**o**) *Chromis chromis*, (**p**) *Symphodus tinca*, (**q**) *Symphodus mediterraneus*, (**r**) *Symphodus cinereus*, (**s**) *Coris julis*, (**t**) *Thalassoma pavo*, (**u**) *Serranus cabrilla*, (**v**) *Epinephelus marginatus*, (**w**) *Sciaena umbra*, (**x**) *Seriola dumerili*, (**y**) *Trachurus* sp., (**z**) *Apogon* sp., (**a.a**) *Atherina* sp., (**a.b**) *Conger conger*, (**a.c**) *Scorpaena* sp., and (**a.d**) Unknown fish.

All time-lapse images were saved with the filename indicating the date (i.e., the year, the month, and the day), the timestamp in Universal Time Coordinates (UTC) (i.e., hour, minutes and seconds), the name of the platform, and finally the camera used for the acquired image^48^. As a result, we had an inspected dataset of 33805 images, depicting a total of 69917 manually tagged fish specimens, 36777 of which pertaining to 29 different taxa (Fig. 4) (Table 5). The remaining specimens (i.e., 33140) were attributed to the unclassified category (see previous section).

**Table 5:** List of fish taxa with their respective number of tags and relative percentage.

Taxa	N	%
*Diplodus vulgaris*	14328	20.49
*Diplodus sargus*	2727	3.90
*Diplodus puntazzo*	374	0.53
*Diplodus cervinus*	415	0.59
*Diplodus annularis*	1268	1.81
*Oblada melanura*	6898	9.87
*Dentex dentex*	615	0.88
*Sparus aurata*	34	0.05
*Sarpa salpa*	208	0.30
*Boops boops*	10	0.01
*Spondyliosoma cantharus*	1001	1.43
*Pagrus pagrus*	50	0.07
*Pagellus* sp.	9	0.01
*Spicara maena*	1826	2.61
*Chromis chromis*	2762	3.95
*Symphodus tinca*	7	0.01
*Symphodus mediterraneus*	209	0.30
*Symphodus cinereus*	54	0.08
*Coris julis*	1589	2.27
*Thalassoma pavo*	53	0.08
*Serranus cabrilla*	258	0.37
*Epinephelus marginatus*	5	0.01
*Sciaena umbra*	50	0.07
*Seriola dumerili*	72	0.10
*Trachurus* sp.	1	0.00
*Apogon* sp.	822	1.18
*Atherina* sp.	101	0.14
*Conger conger*	14	0.02
*Scorpaena* sp.	1017	1.45
Unknown fish	33140	47.40
TOTAL	69917	100.00

Number of tags (N) and relative percentage (%) for each fish taxa, unclassified individuals and total of fishes detected during 2013 and 2014 at the OBSEA platform.

In the dataset file for manual tagging^48^, we reported the timestamp in UTC (yyyy-mm-ddThh:mm:ss) and the filename (e.g., timestamp associated) of the tagged image, plus the fish taxa name and the image vertices’ coordinates of the bounding box (bbox) containing the identified specimens in the OBSEA photo (Fig. 4). In order to improve the reuse of this dataset, we report here its details, described also in the PANGEA repository^48^, in Table 6.

**Table 6:** Details of the dataset with the tags of the fish specimens.

Column Labels	Description
Event	“OBSEA:CAM1:2013_14” if the Sony SNC-RZ25N camera was used to take the photo, or “OBSEA:CAM2:2013_14” if the Axis P1346-E camera was used.
Date/Time	The time stamp information in UTC with “yyyy-mm-ddThh:mm:ss” as format
IMAGE	The image name in the repository that include the time stamp and the type of camera used to take the photo
Species	The species’ Latin name checked with the taxonomy site www.fishbase.org
bboxx1 [pixel]	abscissa value of the first vertex of the tag
bboxy1 [pixel]	ordinate value of the first vertex of the tag
bboxx2 [pixel]	abscissa value of the second vertex of the tag
bboxy2 [pixel]	ordinate value of the second vertex of the tag
bboxx3 [pixel]	abscissa value of the third vertex of the tag
bboxy3 [pixel]	ordinate value of the third vertex of the tag
bboxx4 [pixel]	abscissa value of the fourth vertex of the tag
bboxy4 [pixel]	ordinate value of the fourth vertex of the tag

The details of each variable of the dataset for the manual tagging of the OBSEA photos for the years 2013 and 2014 are reported here, with the timestamp in Universal Time Coordinates (UTC) and the bounding boxes (bbox) coordinates.

The proposed dataset can be used with any image analysis methodology, including the popular Deep Learning (DL) approaches, thanks to the annotated bboxs and related species labels for each fish individual. The bboxs proposed in this work are rotated rectangles that tightly fit each tagged fish individual. Image analysis approaches based on convolutional operators need the bboxs to be rectangles with the edges parallel to the image borders and, depending on the specific implementation, the bboxs could have different encoding. An example is the rectangle encoding for the “You Only Look Once” (YOLO) approach^54^, for which it is very easy to transform the general-purpose rectangle encoding suggested in our work into the YOLO encoding and *vice-versa*.

A recent work on Deep Learning (DL) methods for automatic recognition and classification of fish specimens^55^ identified the paucity of multiple species labelled datasets created by specialists, and with a community-oriented approach as major constraint for this methodology. In our dataset, ground-truthed by specialists, we labelled multiple species of fishes with a great number of tags, and with images taken from a camera focussing the same artificial reef during the whole monitoring period. For this reason, this dataset can be a good material for DL procedures and Artificial Intelligence based approaches in general.

### Oceanographic and meteorological datasets

The measurements from the CTD device of the OBSEA, the meteorological stations of “Development Centre of Remote Acquisition and Information Processing” (SARTI, https://www.sarti.webs.upc.edu/web_v2/) rooftop and the Sant Pere de Ribes station were stored in a PANGEA repository^51–53^. In order to better use this dataset we report the details of these datasets in Tables 7, 8 and 9, respectively.

**Table 7:** Details of the CTD probes measurements’ dataset.

Column Labels	Description
Date/Time	The time stamp information in UTC with “yyyy-mm-ddThh:mm:ss”, as format
Temp [°C]	average value of water temperature
QF Water Temperature	Quality Flag of the water temperature measurement
Temp std dev [±]	standard deviation of the water temperature measurement
Cond [mS/cm]	average value of conductivity
QF conduct	Quality Flag of the conductivity measurement
Cond std dev [±]	standard deviation of the conductivity measurement
Press [dbar]	average value of water pressure
QF water press	Quality Flag of the water pressure measurement
Press std dev [±]	standard deviation of the water pressure measurement
Sal	average value of water salinity
QF sal	Quality Flag of the water salinity measurement
Sal std dev [±]	standard deviation of the water salinity measurement
SV [m/s]	average value of sound velocity
QF SV	Quality Flag of the sound velocity measurement
SV std dev [±]	standard deviation of the sound velocity measurement
Event	“OBSEA:SBE16:2013_14” if the SEA-BIRD SBE16plus V2 SeaCAT device was used for the measurement, or “OBSEA:SBE37:2013_14” if the SEA-BIRD SBE 37-SMP MicroCAT device was used.

The details of each variable of the dataset for the OBSEA CTD probes for the years 2013 and 2014 are reported here with the timestamp in Universal Time Coordinates (UTC).

**Table 8:** Details of the SARTI rooftop meteorological station dataset.

Column Labels	Description
Date/Time	The time stamp information in UTC with “yyyy-mm-ddThh:mm:ss” as format
T air [K]	average value of air temperature
QF air temp	Quality Flag of the air temperature measurement
TTT std dev [±]	standard deviation of the air temperature measurement
ff [m/s]	average value of wind speed
QF wind speed	Quality Flag of the wind speed measurement
ff std [±]	standard deviation of the wind speed measurement
dd [deg]	average value of wind direction
QF wind dir	Quality Flag of the wind direction measurement
PPPP [hPa]	average value of atmospheric pressure
QF atmos press	Quality Flag of the atmospheric pressure measurement
PPPP std [±]	standard deviation of the atmospheric pressure measurement
RH [%]	average value of relative humidity
QF RH	Quality Flag of the relative humidity measurement
RH std [±]	standard deviation of the relative humidity measurement

The details of each variable of the dataset for the “Development Centre of Remote Acquisition and Information Processing” (SARTI) meteorological station for the years 2013 and 2014 are reported here with the timestamp in Universal Time Coordinates (UTC).

**Table 9:** Details of the Sant Pere de Ribes meteorological station dataset.

Column Labels	Description
Date/Time	The time stamp information in UTC with “yyyy-mm-ddThh:mm:ss” as format
E [W/m**2]	value of Irradiance heat flux density measurement
Rain [mm]	value of rainfall measurement

The details of each variable of the dataset for the Sant Pere de Ribes meteorological station for the years 2013 and 2014 are reported here with the timestamp in Universal Time Coordinates (UTC).

Environmental data had temporal gaps in their time series due to sensor malfunction or power/communications loss. The temporal coverage for each variable is detailed in Table 10.

**Table 10:** Temporal coverage of the different environmental data.

Station	Variable	Temporal Coverage (%)
OBSEA	*sea water temperature*	*93.49*
OBSEA	*sea water electrical pressure*	*93.49*
OBSEA	*sea water salinity*	*89.74*
UPC	*air temperature*	*94.68*
UPC	*wind speed*	*94.68*
UPC	*wind direction*	*94.68*
St Pere	*solar irradiance*	*75.77*
St Pere	*rain intensity*	*51.42*

Temporal coverage as percentage (%) for the environmental data acquired at the OBSEA, and at the meteorological stations on the Polytechnic University of Catalonia (UPC) building in Vilanova i la Geltrù and in Sant Pere de Ribes during 2013 and 2014.

## Technical Validation

The manual tagging fish classification was performed following the FishBase website^48^, consulting local fish faunal guides^56–58^. The operator that carried out the tagging trained in the fish classification using the Citizen Science tool of the OBSEA website (https://www.obsea.es/citizenScience/). Furthermore, to better classify the recognizable fish specimens we cross-checked our fish identification with specialists in fish classification from the Institut de Ciències del Mar of Barcelona (ICM-CSIC, www.icm.csic.es).

**Fig. 5:**
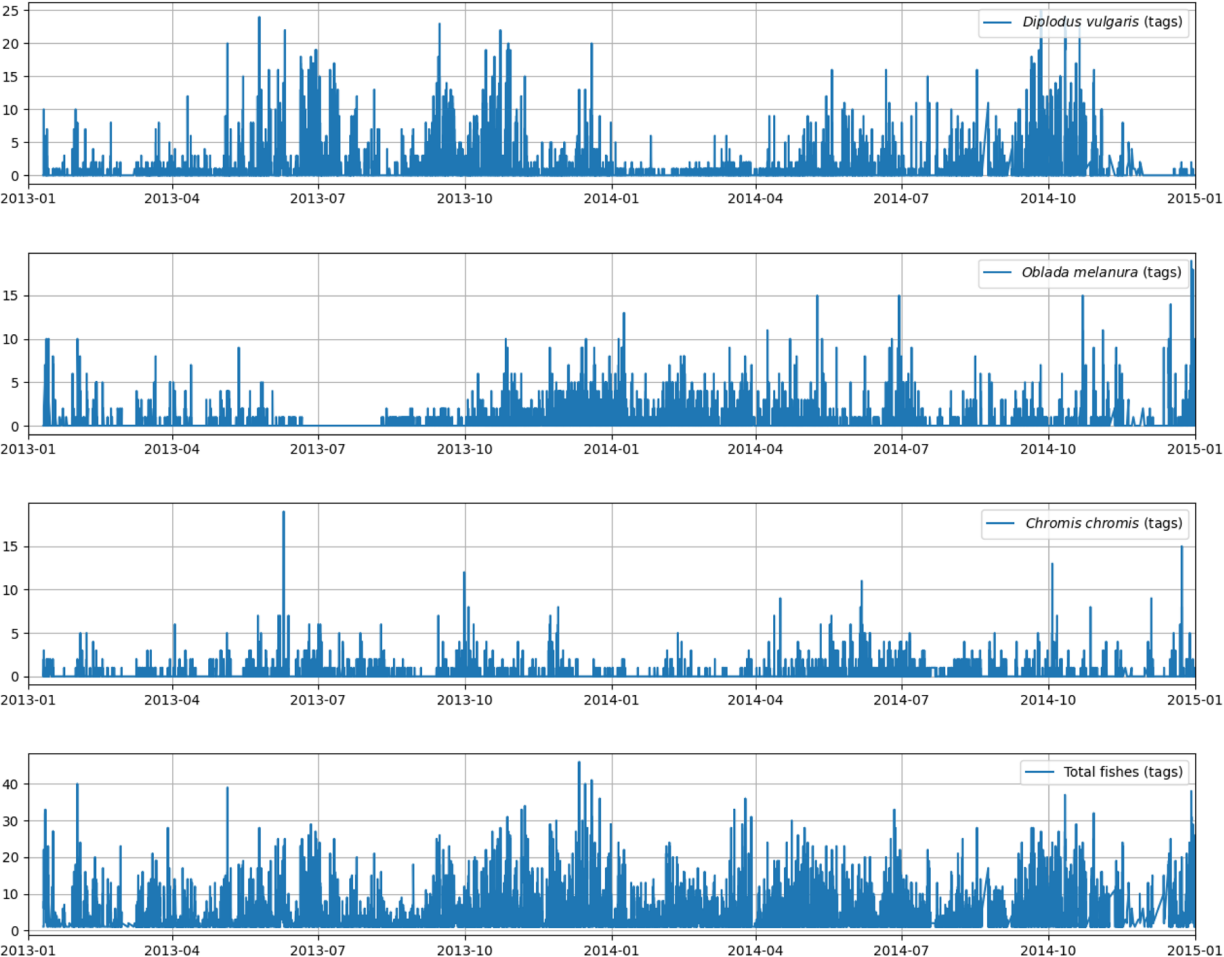
Time series plots of fish individuals. Here we report the time series for the 3 most abundant species (i.e., *Diplodus vulgaris, Oblada melanura*, and *Chromis chromis*) and total of individuals for the tagged fishes at the OBSEA platform between 2013 and 2014.

Here, we report the time series for the three most abundant fish taxa (i.e., *Diplodus vulgaris, Oblada melanura* and *Chromis chromis*) and total fish counts detected during the tagging procedure in order to ensure that there are not large gaps in the image acquisition at the OBSEA during 2013 and 2014, and that the data encompass all the seasons to detect and classify the highest number of species of the local changing fish community (Fig. 5).

**Fig. 6:**
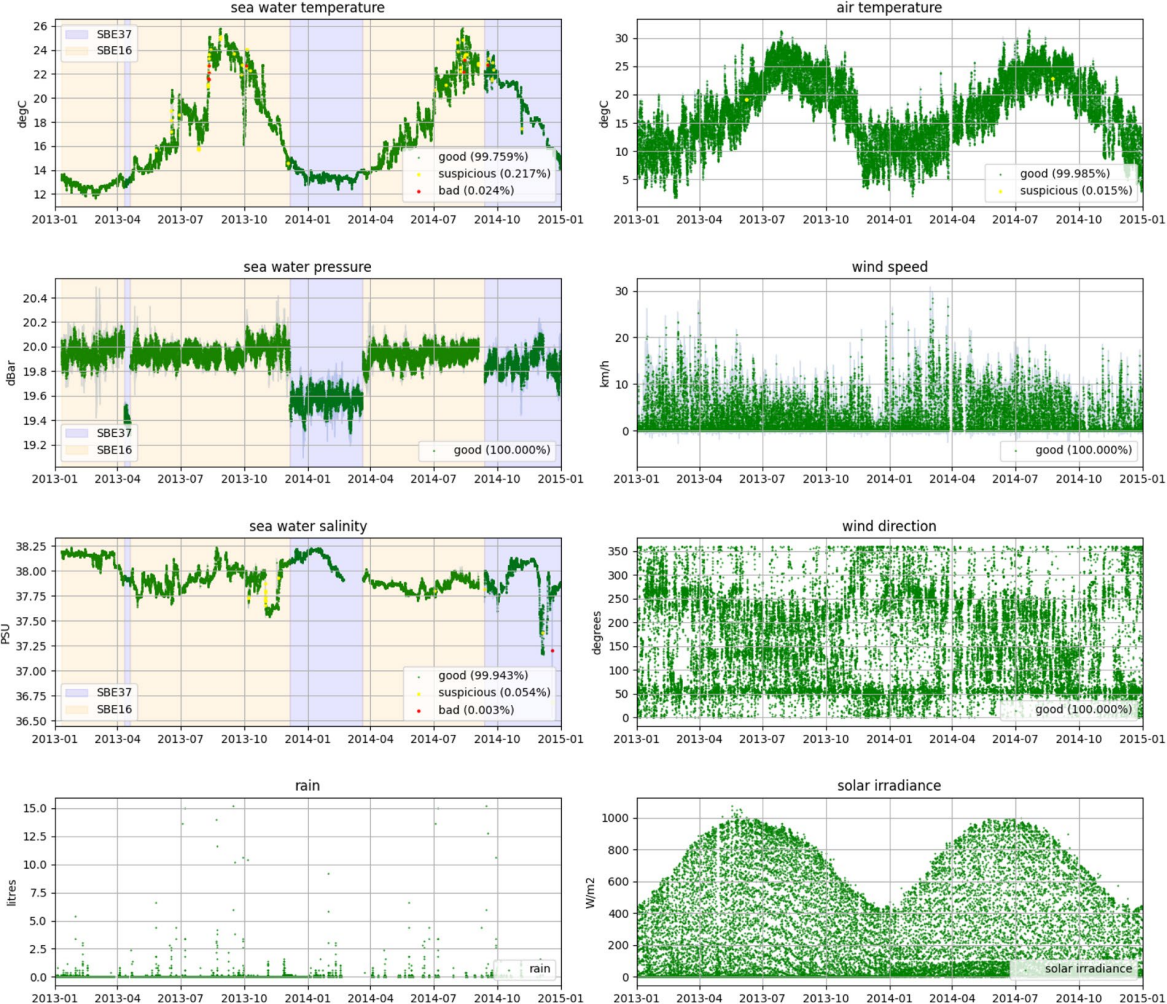
Time series plots of the environmental variables. Here we report the time series for the three oceanographic variables (i.e., water temperature, salinity and depth), and the five meteorological variables (i.e., air temperature, wind speed and direction, solar irradiance and rain) at the OBSEA platform, and meteorological stations on the “Development Centre of Remote Acquisition and Information Processing” (SARTI) rooftop and in Sant Pere de Ribes between 2013 and 2014. In the seawater temperature, pressure and salinity graphs we highlighted the use of SBE37 CTD probe with grey bands, and the SBE16 CTD probe with light yellow bands. The green points in the time series are the good quality data, the yellow ones the suspicious and the red ones the bad. Relative percentage of each QC Indexes was reported in the time series, except for rain and solar irradiance data, considered a prefiltered and institutional source (see previous section).

We also reported the time series of the environmental variables measured at the OBSEA platform, and at the two different meteorological stations on the “Development Centre of Remote Acquisition and Information Processing” (SARTI) rooftop and in Sant Pere de Ribes between 2013 and 2014. These time series are displayed with their respective Quality Control (QC) Indexes highlighted by different colours, in order to ensure the good quality of these data and show the low occurrence of gaps in the time series (see previous section) (Fig. 6).

**Fig. 7:**
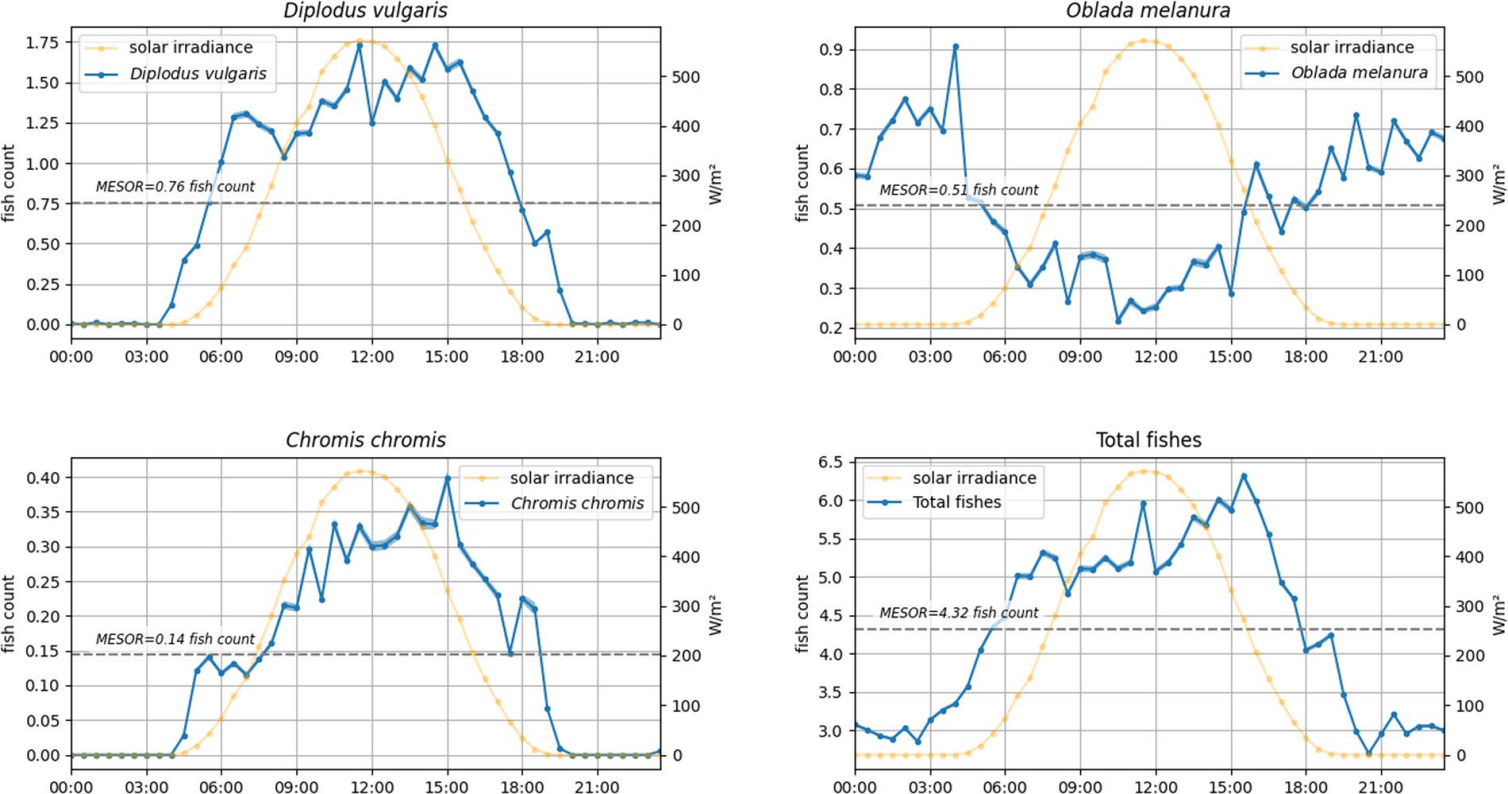
Waveform analysis plots. We reported here the waveforms of the 3 most abundant species (i.e., *Diplodus vulgaris, Oblada melanura*, and *Chromis chromis*) and total of fishes at the OBSEA platform during 2013 and 2014 for the tagged fishes (blue line) related to the photoperiod (yellow line).

As a result, we also show here the resulting graphs from the diel waveform analysis of the tagging data for the three most abundant species and the total number of individuals of fishes related to the solar irradiance respective values to identify the phase of rhythms (i.e., the peak averaged timing as a significant increase in fish counts) in relation to the photoperiod (solving *via* data averaging the problems of gaps in data acquisition) (Fig. 7).

It can be observed that in general the species are diurnal as reported in literature^59^. The only exception is *O. melanura* that was observed more active during crepuscular hours^59^, but in our case was tagged more during nighttime. This could be explained by the better visualisation of this species with illumination, lacking of well recognizable marks for its classification. Therefore, it could be inferred that, in general, the tags for the different species are proportional to the local abundances, except for the certain species, such as *O. melanura*. This last statement is based on a recent article^60^ describing a method for the estimation of organisms’ abundance from visual counts with cameras. The article proposes a Bayesian framework that, under appropriate assumptions, allows to estimate the animals’ density in a single survey without the need to track the movement of the single specimens.

## Usage Notes

As can be observed in Table 5 the classes of the inspected dataset are imbalanced (e.g., there are 14328 *Diplodus vulgaris* tags and only 1 *Trachurus* sp. tag). This characteristic has to be managed by applications dealing with Artificial Intelligence for the automated interpretation of the image content. In case the image analysis method could not manage unbalanced datasets^61,62^, data augmentation approaches could be used for generating new reliable individuals starting from the classes tagged in the dataset^63–65^.

## Data Availability

The developed Python code for tagging and labelling the images is available through the Zenodo repository^49^. Another device that can be used for tagging fishes is the public Label Image tool (https://github.com/tzutalin/labelImg).
